# Immune Cell Infiltration as Signatures for the Diagnosis and Prognosis of Malignant Gynecological Tumors

**DOI:** 10.3389/fcell.2021.702451

**Published:** 2021-06-17

**Authors:** Qi-Fang Liu, Zi-Yi Feng, Li-Li Jiang, Tong-Tong Xu, Si-Man Li, Kui-Ran Liu

**Affiliations:** ^1^Department of Obstetrics and Gynecology, Shengjing Hospital of China Medical University, Shenyang, China; ^2^Department of Plastic Surgery, The First Hospital of China Medical University, Shenyang, China

**Keywords:** diagnosis, malignant gynecological tumors, immune cell, prognosis, biomarker, survival analysis

## Abstract

**Background** Malignant gynecological tumors are the main cause of cancer-related deaths in women worldwide and include uterine carcinosarcomas, endometrial cancer, cervical cancer, ovarian cancer, and breast cancer. This study aims to determine the association between immune cell infiltration and malignant gynecological tumors and construct signatures for diagnosis and prognosis.

**Methods** We acquired malignant gynecological tumor RNA-seq transcriptome data from the TCGA database. Next, the “CIBERSORT” algorithm calculated the infiltration of 22 immune cells in malignant gynecological tumors. To construct diagnosis and prognosis signatures, step-wise regression and LASSO analyses were applied, and nomogram and immune subtypes were further identified.

**Results** Notably, Immune cell infiltration plays a significant role in tumorigenesis and development. There are obvious differences in the distribution of immune cells in normal, and tumor tissues. Resting NK cells, M0 Macrophages, and M1 Macrophages participated in the construction of the diagnostic model, with an AUC value of 0.898. LASSO analyses identified a risk signature including T cells CD8, activated NK cells, Monocytes, M2 Macrophages, resting Mast cells, and Neutrophils, proving the prognostic value for the risk signature. We identified two subtypes according to consensus clustering, where immune subtype 3 presented the highest risk.

**Conclusion** We identified diagnostic and prognostic signatures based on immune cell infiltration. Thus, this study provided a strong basis for the early diagnosis and effective treatment of malignant gynecological tumors.

## Introduction

Malignant gynecological tumors are the main cause of cancer-related death in women worldwide. Typically, common malignant gynecological tumors, including uterine carcinosarcomas, endometrial, cervical, and ovarian cancer and breast cancer, are also considered (Fahad Ullah, 2019). These cancers are closely related to reproductive factors and share common characteristics, suggesting similar etiological pathways or mechanisms (Kelsey et al., 1993; Bates and Bowling, 2013). Breast cancer surpassed lung cancer among all the cancer types to become the most frequently diagnosed cancer and cause of mortality. Moreover, the mortality of other female reproductive cancers should not be underestimated (Sung et al., 2021). Thus, it is of great significance to determine the effective biomarkers for promoting the diagnosis and prognosis of patients with these cancers.

The main treatments of malignant gynecological tumors include surgery, chemotherapy, and radiotherapy (Denschlag and Ulrich, 2018; Chandra et al., 2019; Koh et al., 2019; Rossi et al., 2019). Among them, radical surgery is usually the intervention of choice. Chemotherapy and radiotherapy have also been performed as adjuncts to surgery, for reducing the size of tumors and ameliorating their recurrence (Wang et al., 2011; Bestvina and Fleming, 2016; Matei et al., 2019). Occasionally, local palliative treatments are necessary for alleviating the pain that patients experience (Davidson et al., 2018).

Nevertheless, many needs remain unaddressed; advanced stage diseases are still incurable, with numerous patients dying of gynecological tumors annually. With the deepening of the research on the immune system, immunotherapy has become a very promising treatment method that can be used after surgery and chemotherapy. Different immunotherapy strategies are adopted for different categories of immunocompromised patients. However, complications such as specific antigen recognition and the treatment of adverse reactions remain unresolved (Tagliabue et al., 2018). Developing methods to improve toxicity to cancers, identify more specific targets, and improve their efficacy and safety are the difficulties we must overcome (Pandolfi et al., 2018).

Recently, the use of immunotherapies to treat cancer patients has become a reality (Gajewski et al., 2013). More studies are increasingly focused on the tumor microenvironment, which can act as potential biomarkers to increase the accuracy of diagnoses and prognoses and provide opportunities for new cancer therapy strategies (Masugi et al., 2019; Yang et al., 2019). The infiltrating immune cells are an essential part of the tumor microenvironment and may exhibit tumor-antagonizing or tumor-promoting effects (Wang et al., 2019; Lei et al., 2020). While the immune microenvironment was analyzed in various cancer studies (Stanton and Disis, 2016; Karn et al., 2017; Zhang et al., 2020), few comprehensively analyze the role of immune cell infiltration in malignant gynecological tumors.

CIBERSORT (Cell-Type Identification by Estimating Relative Subsets of RNA Transcripts) is a new algorithm for calculating the quantity of immune cells. It contains 547 genes and 22 types of common human immune cells in Newman et al. (2015). Moreover, it can also determine the immune cell landscape of various tumors and select related biomarkers for diagnosis and prognosis (Yang et al., 2019). Much research has been carried out with CIBERSORT to study the tumor microenvironment (Blum et al., 2018) further.

Our study estimated the proportion of 22 immune cells in malignant gynecological tumors based on the CIBERSORT algorithm using the sample expression data downloaded from TCGA. We further constructed the diagnosis and prognosis models, which provided a strong basis for early diagnosis and effective treatment of malignant gynecological tumors.

## Materials and Methods

### Data Acquisition

The data used in the study were all obtained from open-source databases. The cohort of the female reproductive system used to determine the immune signature consisted of endometrial, uterine, ovarian cancer, cervical, and breast cancer data. For more comprehensive results, female breast cancer data were also included. We retrieved all RNA-seq transcriptome cancer data from The Cancer Genome Atlas (TCGA) database^1^ (Blum et al., 2018).

Due to the shortage of normal samples in the TCGA database, data from the GTEx database (mainly from autopsies) were selected to expand the subset of normal data samples^2^. Then, the RNA-seq transcriptome data were normalized by fragment per kilobase of exon model per million (FPKM, mean fragment per kilobase million). The exact sample number, data sources, and primary organs are listed in Table 1, and a total of 2,562 data samples and 25,496 genes were obtained.

**TABLE 1:** Samples’ basic characteristics.

**Item**	**Tumor sample (*n* = 2,013)**	**Percent (%)**	**Normal sample (*n* = 494)**	**Percent (%)**
**Cancer type**				
UCEC	181	9	101	20.45
CESC	306	15.20	13	2.63
OV	427	21.21	88	17.81
BRCA	1,099	54.60	292	59.11
**Diagnosis analysis**				
Training cohort	1,007	50	247	50
Validation cohort	1,006	50	247	50
**Prognosis analysis**				
Training cohort	1,127	70		
Validation cohort	604	30		

Furthermore, we downloaded the patients’ clinicopathological information which consisted of their age, gender, survival time, outcome, and TNM stage from the TCGA database with the approval of the TCGA. The samples with missing or incorrect follow-up data and less than 30 days follow-up time were removed and excluded from the prognostic analysis; however, they were included in the diagnostic analysis.

### Analysis of Infiltrating Immune Cell Components

To estimate the immune cell components in each sample, CIBERSORT^3^ was used with the LM22 signature and 1,000 permutations (Newman et al., 2015). We used a panel of 22 immune cells consisting of B cells, T cells, natural killer cells, macrophages, dendritic cells, and myeloid subsets. CIBERSORT acquires a probability, P for the deconvolution of each sample via Monte Carlo sampling, providing a measure of confidence in the results. In our analysis, *P* < 0.05 means the results calculated by the CIBERSORT are accurate, subsequently, only 506 samples (*P* < 0.05) were used in the follow-up analysis. The final output estimates were normalized for each sample, and the summary of each immune cell component was 1.

### Diagnostic Analysis

The diagnostic analysis was carried out among the eligible samples, which were randomly split into training and validation cohorts with a 5:5 ratio using the R package “caret”^4^. Logistic regression was used to construct the diagnostic signature of the training group, and step-wise regression was used to screen the variables. Receiver operating characteristic (ROC) curves were used to analyze the predictive efficacy of the signatures, and the area under the curve (AUC) was calculated. This result was further tested and verified in the training cohort, the validation cohort, and for all datasets.

### Prognostic Analysis

Only the samples that met the inclusion criteria with complete clinical and follow-up information were included in the prognostic analysis. The eligible patients were separated into training and validation cohorts in a 7:3 ratio using the R package “caret,” and then the LASSO analysis was conducted to obtain a predictive signature from the training cohort. The coefficients characterized the risk score according to the least absolute shrinkage and selection operator (LASSO) algorithm by using the R package “glmnet”^5^. A risk score was calculated by applying the following formula (Huang et al., 2020):risk score =

$$\sum_{i=1}^{n}\text{C}od\text{f}i^{*}{\text{x}}_{i}$$

where *Codfi* is the coefficient and **x_i_** is the relative expression value of each of the candidate immune cells. The samples in the training- and validation- groups were divided into high- and low-risk groups, and the median risk score was used as the cut-off point. A Kaplan–Meier analysis was conducted to assess the difference in overall survival between the training set, validation set, and datasets.

### Validation of Diagnostic Signature and Prognostic Signature in Geo Datasets

We constructed other cohorts from Gene Expression Omnibus (GEO) to further demonstrate the effectiveness of the diagnostic signature and prognostic signature. These cohorts were selected with a search scope limited to “*Homo sapiens*,” and the chip platform limited to GPL57, GPL7759, and other common platforms. Furthermore, the cohort that met the following exclusion criteria was not selected: (i) datasets that used cell lines or animal samples; (ii) the patients’ survival information was not complete. After confirmation, CIBERSORT was again used to confirm the immune components, followed by verification of the reliability and validity of the diagnostic, and prognostic signatures.

### Nomogram Construction

Nomograms are simplified models for predicting the cancer prognosis as a single numerical value. The length of the line represents the indicator’s impact on the results, and a longer line represents a greater impact. The nomogram application is achieved by adding together all the point scales of each variable. The total points projected on the bottom scales represent the probability of 3-year, and 5-year overall survival. The R package “rms”^6^ was used to draw the nomogram, and the R package “survivalROC” was to compile the ROC curve.

### Identification of Immune Subtypes

We performed an unbiased grouping of all patients using consensus clustering analysis with the R package “ConsensusClusterPlus”^7^ to explore the correlation between different immune cell infiltration subtypes and the prognosis of patients. In addition, we conducted a survival analysis of various immune subtypes.

### Statistical Analysis

R software (Version 4.0.3) was used for all statistical analyses, and the data were shown as mean ± standard deviation. The default Wilcoxon test and one-way analysis of variance (ANOVA) were used to analyze the differences between the two groups and among multiple groups, respectively. The overall differences in survival rate among groups were quantified via Kaplan–Meier analysis and a log-rank test. Results were regarded statistically significant when *P* < 0.05.

## Results

### Patient Characteristics

**FIGURE 1:**
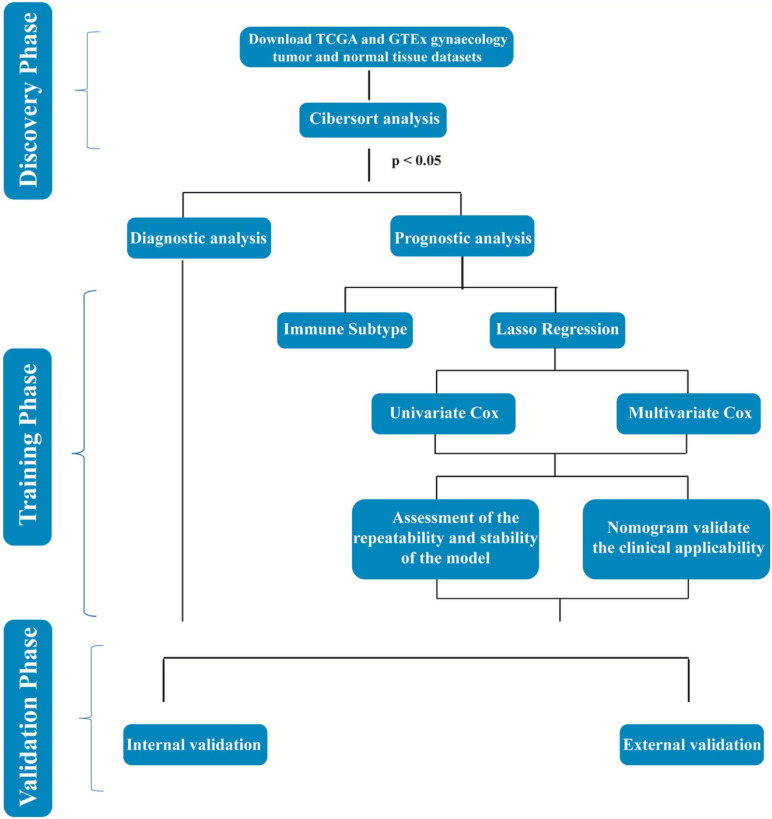
Workflow chart of data generation and analysis.

Immune cell infiltration is necessary for the initiation and progression of cancer. We developed selection criteria to assess the biological role of immune cell infiltration in malignant gynecological tumors and downloaded them from the TCGA database and GTEx database. The resulting *P* < 0. 05 samples in CIBERSORT were used for further analysis. In total, 2,057 patients were diagnosed with female reproductive system tumors (181 UCEC samples, 306 CESC samples, 427 OV samples, and 1,099 BRCA samples), and 494 normal samples were selected. The detailed distribution of the patients in each group is summarized in Table 1, and the workflow of the study is illustrated in Figure 1.

### Composition of Immune Cells in Malignant Gynecological Tumors

**FIGURE 2:**
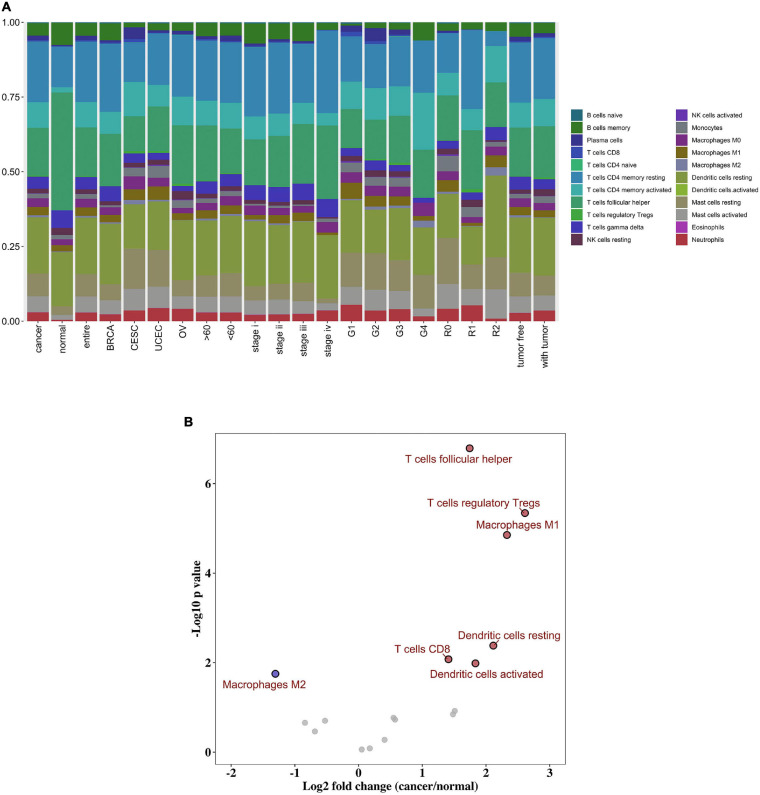
Composition of immune cells in gynecological malignant tumors. **(A)** Summary of inferred immune cell subsets. **(B)** Volcano plot visualized the differentially infiltrated immune cells between tumor tissues and normal tissues. Red represents up-regulated, while blue represents down-regulated.

The distribution of the immune cells in and across clinical groups of the malignant gynecological tumors is shown in Figure 2A. We can deduce that the five most common immune cell fractions were follicular helper T cells, activated CD4 memory T cells, CD4 memory resting T cells, resting Dendritic cells, and resting mast cells. The total proportion of the five immune cells were more than 60% in all clinical subgroups (Supplementary Figure 1).

However, in normal tissue, follicular helper T cells, resting Dendritic cells, resting CD4 memory T cells, memory B cells, and gamma delta T cells were the five main immune cells; and their total proportion surpasses 70%. In addition, we further distinguished the discrepancy between each immune cell within tumor, and normal tissues. As shown in Figure 2B, the follicular helper T cells, activated CD4 memory T cells, CD4 memory resting T cells, resting Dendritic cells, and resting mast cells were all up-regulated in the cancer group, while the M2 Macrophages were down-regulated. Here, *P* < 0.05 was considered to be a statistically significant result (Supplementary Table 1).

### Diagnostic Signature Building

**FIGURE 3:**
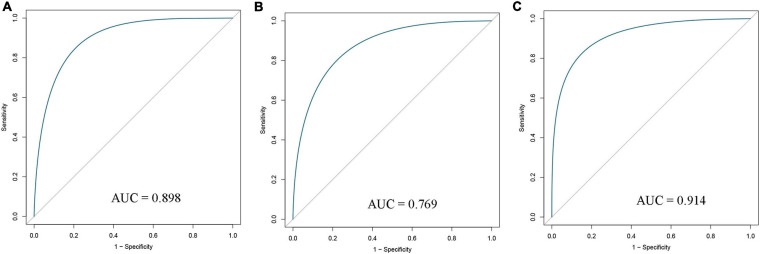
Receiver operating characteristic (ROC) curves of diagnostic signature in the training **(A)**, validation **(B)**, and entire **(C)** cohorts. AUC, area under ROC curve.

All selected samples were spilt into a training cohort (1,007 samples) and a validation cohort (1,006 samples). A logistic regression model was built based on the training set, and variables were screened using step-wise regression (see Supplementary Table 2). We observed that the resting NK cells, M0 Macrophages, and M1 Macrophages all satisfied the condition that *P* < 0. 05. Thus, they were chosen as variables for building the diagnostic signature. We also predicted that the results of the tumor and normal tissues in the training, validation, and entire cohorts to further verify the diagnostic value of our model. The ROC curve suggested that our model had high accuracy (AUC = 0.898, 0.769, and 0.914, respectively; Figures 3A–C).

### Prognostic Signature Building

**FIGURE 4:**
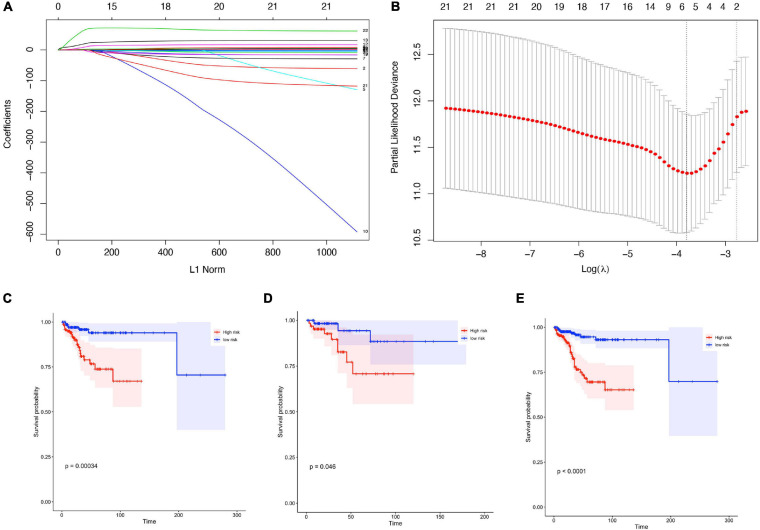
Construction of prognostic signature in patients with gynecological malignant tumors. **(A)** Least absolute shrinkage and selection operator (LASSO) coefficient profiles of the fractions of 22 immune cell types. **(B)** Tenfold cross-validation for tuning parameter selection in the LASSO model. **(C–E)** Kaplan-Meier curves for survival in the training cohorts **(C)**, validation cohort **(D)**, entire cohort **(E)**.

Based on our screening criteria, 1,731 patients with over 30 days follow-up time were first distributed randomly into the training cohort (1,127 samples) and validation cohort (604 samples) at a 7:3 ratio. Next, it was used to construct the prognostic signature using LASSO-Cox analysis (Figures 4A,B). Six important immune cells were identified, including CD8T cells, activated NK cells, Monocytes, M2 Macrophages, resting Mast cells, and Neutrophils (Supplementary Table 3).

The training cohort’s risk scores were then estimated using the LASSO algorithm coefficients. The formula was as follows: risk score = (−4.638 ^∗^ expression level of B cells naive) + (−0.259 ^∗^ expression level of T cells CD8) + (11.463 ^∗^ expression level of NK cells activated) + (22.048 ^∗^ expression level of Monocytes) + (2.841 ^∗^ expression level of M2 Macrophages) + (−4.073 ^∗^ resting Mast cells) + (68.399 ^∗^ expression level of Neutrophils). The training group samples were then split into high- and low-risk groups, and the median value was used as the dividing line. The Kaplan-Meier curves were assessed to ensure that patients scoring as high-risk had a higher survival possibility in the training cohort (Figure 4C).

To ensure the prognostic model’s consistency in predicting results in different groups, we used the same formula to calculate risk factors and for validation of the whole cohorts. Median risk scores were also treated as the cut-off value for distinguishing between the high- or low-risk groups, and the results were consistent with those in the training cohort. A higher risk score corresponded to short survival probability in both the validation cohort (*P* = 0.046, Figure 4D) and the entire cohort (*P* < 0.0001, Figure 4E).

### Validation of the Diagnostic Signature and Prognostic Signature Using the GEO Datasets

The following datasets: GSE21422+GSE42568 (BRCA), GSE54388 (OV), GSE54388+GSE14407 (OV), and GSE63514 (CESC) were downloaded from the GEO database to test the value of the diagnostic signature (Supplementary Table 4). In each group, there was a high diagnostic accuracy for the tumor samples; subsequently, the AUCs were 0.8523, 0.83, 0.67, and 0.71, respectively.

Furthermore, the GSE20685 (BRCA), and GSE53963 + GSE32062 (OV) datasets were both treated as a group to verify the prognostic value of our signature (Supplementary Table 5). Consistent with our TCGA database results, the higher risk scores represented a lower possibility of survival in patients. However, the result showed a notable difference in BRCA; here, patients with a high-risk score experienced good survival. Thus, as mentioned above, both results were statistically significant.

### Multivariate Cox Regression Analyses

To test the clinical indicators, a multivariate Cox model was constructed for the training, internal validation, and full data sets to estimate whether clinicopathological characteristics (including age, tumor stage, cancer status, residual tumor, and tumor grade) could be independent prognostic factors in malignant gynecological tumors (Table 2). In this multivariate analysis, the tumor stage and cancer status influenced all data sets (HR > 1, *P* < 0.05), so they were selected as effective clinical indicators for further analysis.

**TABLE 2:** Multivariable Cox regression analysis of prognosis signature in different cohorts.

	**Training cohort**		**Validation cohort**		**Entire cohort**	
	**Hazard ratio**	***p*-value**	**Hazard ratio**	***p*-value**	**Hazard ratio**	***p*-value**
**Age**						
<60	1.00 (reference)		1.00 (reference)		1.00 (reference)	
>60	1.525 (0.733–3.172)	0.261	1.479 (0.515–4.246)	0.467	1.495 (0.83–2.691)	0.18
**Tumor stage**						
I, II	1.00 (reference)		1.00 (reference)		1.00 (reference)	
III, IV	2.947 (1.335–6.502)	0.007	2.672 (0.977–7.309)	0.056	2.869 (1.543–5.336)	<0.001
**Cancer status**						
Tumor-free	1.00 (reference)		1.00 (reference)		1.00 (reference)	
With tumor	6.012 (2.507–14.418)	<0.001	5.394 (1.573–18.494)	0.007	5.714 (2.821–11.572)	<0.001
**Residual tumor**						
R0	1.00 (reference)		1.00 (reference)		1.00 (reference)	
R1 + R2	0.83 (0.286–2.414)	0.733	NA	NA	1.298 (0.459–3.667)	0.623
**Tumor grade**						
G1 + G2	1.00 (reference)		1.00 (reference)		1.00 (reference)	
G3	1.476 (0.614–3.547)	0.384	1.585 (0.517–4.861)	0.42	1.521 (0.765–3.026)	0.232

### Identification of the Nomogram

**FIGURE 5:**
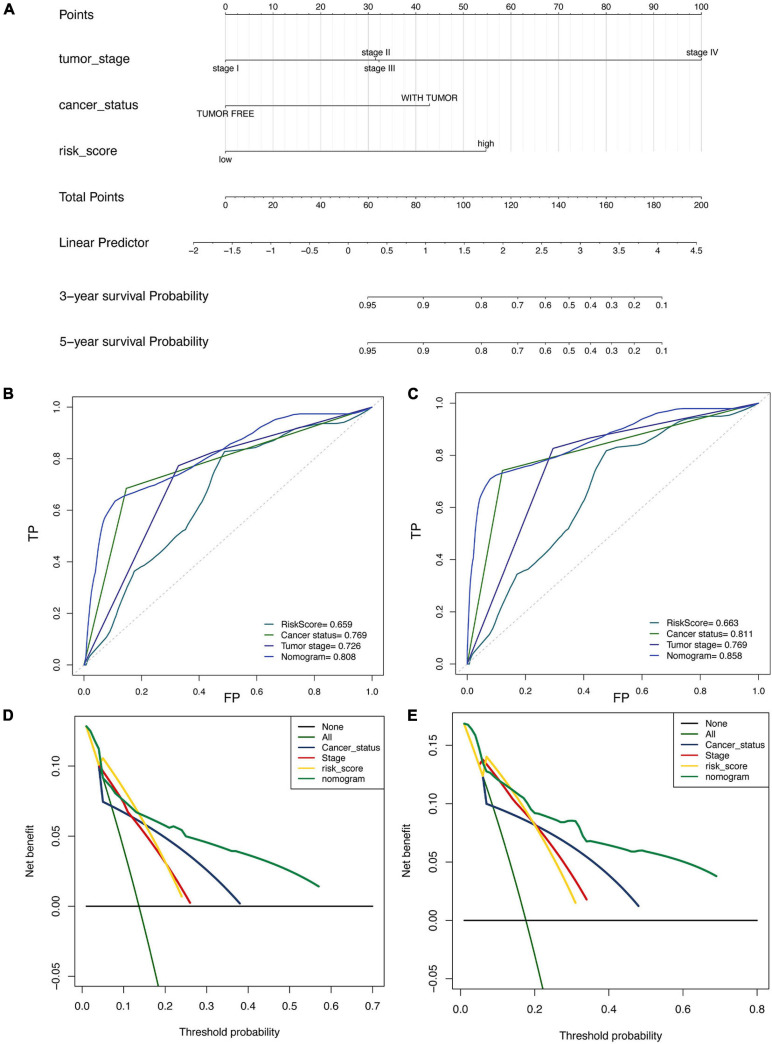
Construction and validation of nomogram in patients with gynecological malignant tumors. **(A)** Nomogram integrated risk factors such as risk signature, age and stage. **(B,C)** The ROC curve for 3-year **(B)** and the 5-year **(C)**. **(D,E)** Decision curve analysis for 3-year **(D)** and the 5-year **(E)**.

A prognostic nomogram based on clinical information was constructed to produce a quantitative method for predicting the prognosis of patients with malignant gynecological tumors. The nomogram (Figure 5A) integrated risk factors such as risk signature, age, and stage, and the results indicated that the tumor stage had the greatest impact on the model. The later tumor stage indicated a lower survival rate in patients, while patients with higher “with tumor” and “risk score” had a higher risk of a poor prognosis.

Moreover, the 3-year (Figure 5B) and 5-year (Figure 5C) ROC curve directly showed that the value of the risk factors. The nomogram had the highest accuracy, when the areas under the ROC curve (AUC) were 0.808 and 0.858. The decision curve analysis (Figures 5D,E) showed similar results, indicating that the nomogram has proper clinical applicability.

### Immune Subtypes

We grouped all 1,731 malignant gynecological tumor cases in an unbiased way to discriminate clear types of immune infiltration by using consensus clustering analysis. The stability of the clustering increased from *k* = 2–10 (Supplementary Figure 2), and *K* = 5 was considered the most optimal choice, so five immune subtypes were determined. Furthermore, the relevance between various cancers and immune subtypes is exhibited in Table 3. BRCA patients were primarily distributed in the immune subtypes 1 and 4, while UCEC patients were mostly distributed in immune subtype 5. Nearly half of the OV patients were distributed in immune subtype 3, while CESC patients were mainly distributed in both immune subtypes 2 and 5.

**TABLE 3:** Relationship between cancer types and immune subtypes.

**Immune subtype**	**BRCA**	**UCEC**	**OV**	**CESC**	**Total (n)**
1	70	1	4	6	81
2	47	8	3	36	94
3	37	3	23	5	68
4	98	7	16	3	124
5	20	13	12	32	77
Total (n)	272	32	58	82	444

**FIGURE 6:**
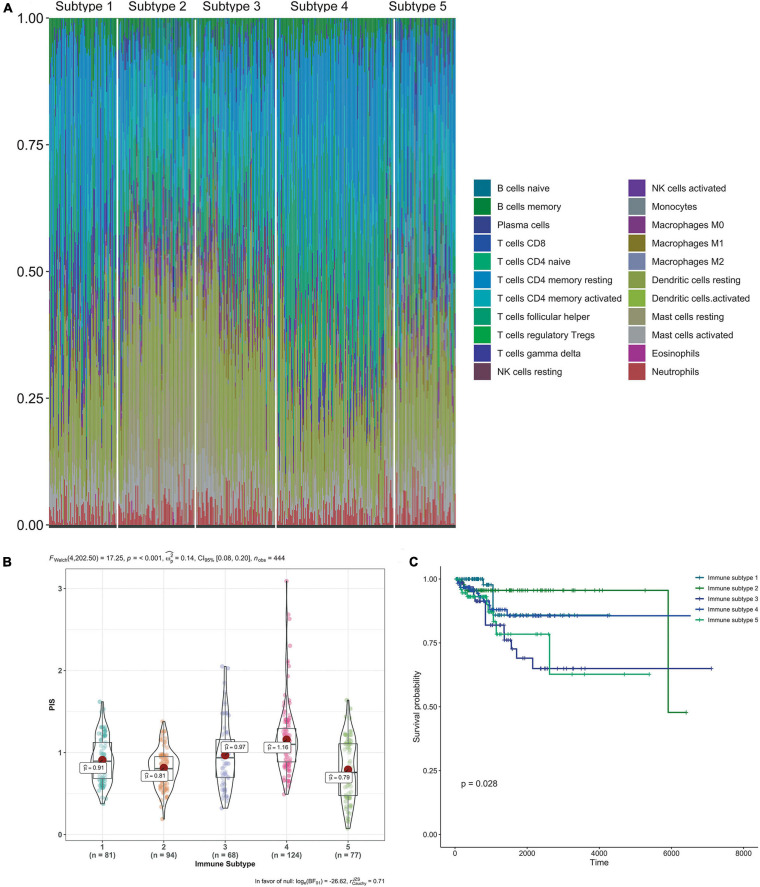
Immune subtypes in patients with gynecological malignant tumors. **(A)** Unsupervised clustering of all samples based on immune cell proportions. Stacked bar charts of samples ordered by cluster assignment. **(B)** Risk score in different immune subtypes. **(C)** Survival analysis of patients within different immune subtypes.

Each immune cell’s specific distribution in each immune subtype is exhibited in Figure 6A. Among them, immune subtype 1 was characterized by high levels of resting CD4 memory T cells, while immune subtype 2, immune subtype 3, and immune subtype 5 were defined by resting dendritic cells and activated dendritic cells. Immune subtype 4 was defined by both resting and activated CD4 memory T cell types.

Also, the calculated risk scores for different subgroups (Figure 6B) indicate that the immune subtypes 3 and 4 had significantly higher risk scores than the other subtypes. Combined with the risk score distribution and Kaplan–Meier analysis (Figure 6C), immune subtype 3 was the most high-risk subtype.

## Discussion

Gynecological cancer is both the most common cancer in women and the leading cause of death in women. The currently treatment methods used, include surgery, radiotherapy, and chemotherapy, are gradually improving. In recent years, immunotherapy research has steadily expanded, and the research results are constantly being applied in clinical practice. However, due to untimely diagnoses and tumor invasiveness, the survival rate of advanced patients is still exceptionally low. Therefore, it is necessary to construct new and effective diagnosis or prognosis signatures for early diagnosis and to improve treatment methods.

Notably, recent developments in novel cancer treatment modalities have focused primarily on early intervention. Munoz and Plevritis (2018) presented a predictive model using the estrogen receptor and human epidermal growth factor receptor 2 status to determine potential survival outcomes. Likewise, Chen et al. (2019) used five lncRNAs data in the TCGA database to obtain a five-lncRNA signature for use as an independent risk factor for OC recurrence. Furthermore, research on tumor microenvironments in cancer has gradually become popular. Yang et al. (2019) applied immune cell infiltration in cancers of the digestive system to process an effective diagnostic and prognostic model for these cancer types. Thus, there is a need for a greater mechanistic understanding of immune cell infiltration’s varied role in tumor progression. We attempted to determine how it participates in tumorigenesis, along with the development and prognosis of malignant gynecological tumors.

First, the newly developed CIBERSORT algorithm was used to determine the composition of immune cells in each sample. We found notable differences in the proportion of immune cells between normal samples and tumor samples, different tumors, different age groups, and different stage groups. Based on the differences between the tumor and normal groups, we selected the samples with *p* < 0.05 and then used the step-wise regression model, resting NK cells, M0 Macrophages, and M1 macrophages to develop a structured diagnostic model. The AUC = 0.8981 value indicated that our model was accurate (89.8% of cases) at diagnosing tumors. Moreover, it also proved the immune system’s involvement in the occurrence and development of cancer.

In this article, candidate cells used to build the prognostic model were also applied according to the high-throughput gene expression generated by CIBERSORT. The LASSO-Cox analysis selected the CD8T cells activated NK cells, Monocytes, M2 Macrophages, resting Mast cells, and Neutrophils as the key biomarkers. According to the expression quantity and expression coefficient of the abovementioned cells, we obtained the risk value of each sample and divided it into high-risk and low-risk groups. The Kaplan-Meier curves confirmed that the patients with high-risk scores had a higher possibility of survival in the training cohort. The results of the internal and external verification sets were consistent with the above results. Furthermore, the multivariate Cox prognostic analysis confirmed that the tumor stage and cancer status impacted all data sets and could be used as an independent prognostic factor.

To better understand the prognosis of the patients, we simplified the models to predict cancer prognosis into a single numerical value, as the nomogram. It integrated tumor stage, cancer status, and risk score, along with the compiling 3-year, and 5-year ROC curves. The results showed that the nomogram has good clinical applicability. Reports have demonstrated a connection between the tumor’s immune microenvironment and its survival rate (Li et al., 2017; Anichini, 2019). Based on the abundance of immune cells, five immune subtypes were identified by consensus cluster analysis, and we further explored the distribution of patients among the different immune subtypes. Combined with the risk score distribution and Kaplan–Meier analysis, immune subtypes 3 was identified as the most high-risk subtype.

Many studies have reported the impacts of the tumor microenvironment on the development and prognosis of tumors, including esophageal (Lin et al., 2016), pancreatic (Wei et al., 2019), colorectal (Roelands et al., 2017), and gastric cancers (Lazãr et al., 2018), as well as melanoma (Huang et al., 2020). However, this research provided comprehensive immune profiles of malignant gynecological tumors, and the resulting diagnostic and prognostic models could serve as biomarkers for early diagnoses and therefore the early initiation of treatment, and for predicting survival.

## Data Availability Statement

The datasets presented in this study can be found in online repositories. The names of the repository/repositories and accession number(s) can be found in the article/Supplementary Material.

## Author Contributions

Q-FL and K-RL: conceptualization. Z-YF and T-TX: original manuscript preparation. L-LJ and S-ML: draft correction, supervision, and editing. All authors listed have made substantial contribution to the manuscript, which was acknowledged and confirmed by themselves, and read and agreed on the final version of the manuscript.

## Conflict of Interest

The authors declare that the research was conducted in the absence of any commercial or financial relationships that could be construed as a potential conflict of interest.

## Footnotes

1 http://cancergenome.nih.gov/

2 https://www.gtexportal.org/home/datasets

3 https://cibersort.stanford.edu/

4 https://cran.r-project.org/web/packages/caret/index.html

5 https://cran.r-project.org/web/packages/glmnetcr/index.html

6 https://cran.r-project.org/web/packages/rms/index.html

7 http://www.bioconductor.org/packages/release/bioc/html/ConsensusClusterPlus.html
