# Supplementary material for: Immune Cell Infiltration as Signatures for the Diagnosis and Prognosis of Malignant Gynecological Tumors
Source: Front Cell Dev Biol. 2021 Jun 17;9:702451. doi: 10.3389/fcell.2021.702451 (PMC8247483; doi:10.3389/fcell.2021.702451)
Supplement: Supplementary file 1 [file Table_1.DOCX]

Supplementary Table 1 | The expression difference of infiltrated immune cells between tumor tissues and normal tissues

|  | P | Foldchange |
| --- | --- | --- |
| B cells naive | 0.22 | 0.558 |
| B cells memory | 0.143 | 2.793 |
| Plasma cells | 0.53 | 1.326 |
| T cells CD8 | 0.008 | 2.658 |
| T cells CD4 memory activated | 0.12 | 2.846 |
| T cells follicular helper | < 0.001 | 3.348 |
| T cells regulatory Tregs | < 0.001 | 6.11 |
| NK cells resting | 0.171 | 1.463 |
| Monocytes | 0.345 | 0.622 |
| Macrophages M0 | 0.187 | 1.487 |
| Macrophages M1 | <0.001 | 5.023 |
| Macrophages M2 | 0.018 | 0.404 |
| Dendritic cells resting | 0.004 | 4.331 |
| Dendritic cells activated | 0.01 | 3.566 |
| Mast cells resting | 0.199 | 0.694 |
