# Supplementary material for: Immune Cell Infiltration as Signatures for the Diagnosis and Prognosis of Malignant Gynecological Tumors
Source: Front Cell Dev Biol. 2021 Jun 17;9:702451. doi: 10.3389/fcell.2021.702451 (PMC8247483; doi:10.3389/fcell.2021.702451)
Supplement: Supplementary file 2 [file Table_2.DOCX]

Supplementary Table 2 | Diagnostic model analysis: Stepwise regression model parameters
