# Supplementary material for: Immune Cell Infiltration as Signatures for the Diagnosis and Prognosis of Malignant Gynecological Tumors
Source: Front Cell Dev Biol. 2021 Jun 17;9:702451. doi: 10.3389/fcell.2021.702451 (PMC8247483; doi:10.3389/fcell.2021.702451)
Supplement: Supplementary file 3 [file Table_3.DOCX]

Supplementary Table 3 | Immune cell coefficient in LASSO regression

| Geneids | Index.min |
| --- | --- |
| T cells CD8 | -0.259 |
| NK cells activated | 11.463 |
| Monocytes | 22.048 |
| Macrophages M2 | 2.841 |
| Mast cells resting | -4.073 |
| Neutrophils | 68.399 |
