# Supplementary material for: Immune Cell Infiltration as Signatures for the Diagnosis and Prognosis of Malignant Gynecological Tumors
Source: Front Cell Dev Biol. 2021 Jun 17;9:702451. doi: 10.3389/fcell.2021.702451 (PMC8247483; doi:10.3389/fcell.2021.702451)
Supplement: Supplementary file 4 [file Table_4.DOCX]

Supplementary Table 4 | The validation of the diagnostic signature in GEO datasets

| Cancer type | GEO datasets | Normal sample | Tumor sample | AUC of ROC |
| --- | --- | --- | --- | --- |
| BRCA | GSE21422+GSE42568 | 22 | 118 | 0.8523 |
| OV | GSE54388 | 6 | 16 | 0.83 |
| OV | GSE54388+GSE14407 | 18 | 28 | 0.67 |
| CESC | GSE63514 | 24 | 28 | 0.71 |
