# Supplementary material for: Immune Cell Infiltration as Signatures for the Diagnosis and Prognosis of Malignant Gynecological Tumors
Source: Front Cell Dev Biol. 2021 Jun 17;9:702451. doi: 10.3389/fcell.2021.702451 (PMC8247483; doi:10.3389/fcell.2021.702451)
Supplement: Supplementary file 5 [file Table_5.DOCX]

Supplementary Table 5 | The validation of the prognostic signature in GEO datasets

| Cancer type | GEO datasets | Number | HR (95% CI) | P value |
| --- | --- | --- | --- | --- |
| BRCA | GSE20685 | 318 | 0.64 (0.41-1.00) | 0.049 |
| OV | GSE53963 + GSE32062 | 249 | 1.57 (1.14-2.17) | 0.0064 |
