# Supplementary figures and images for: Immune Cell Infiltration as Signatures for the Diagnosis and Prognosis of Malignant Gynecological Tumors
Source: Front Cell Dev Biol. 2021 Jun 17;9:702451. doi: 10.3389/fcell.2021.702451 (PMC8247483; doi:10.3389/fcell.2021.702451)

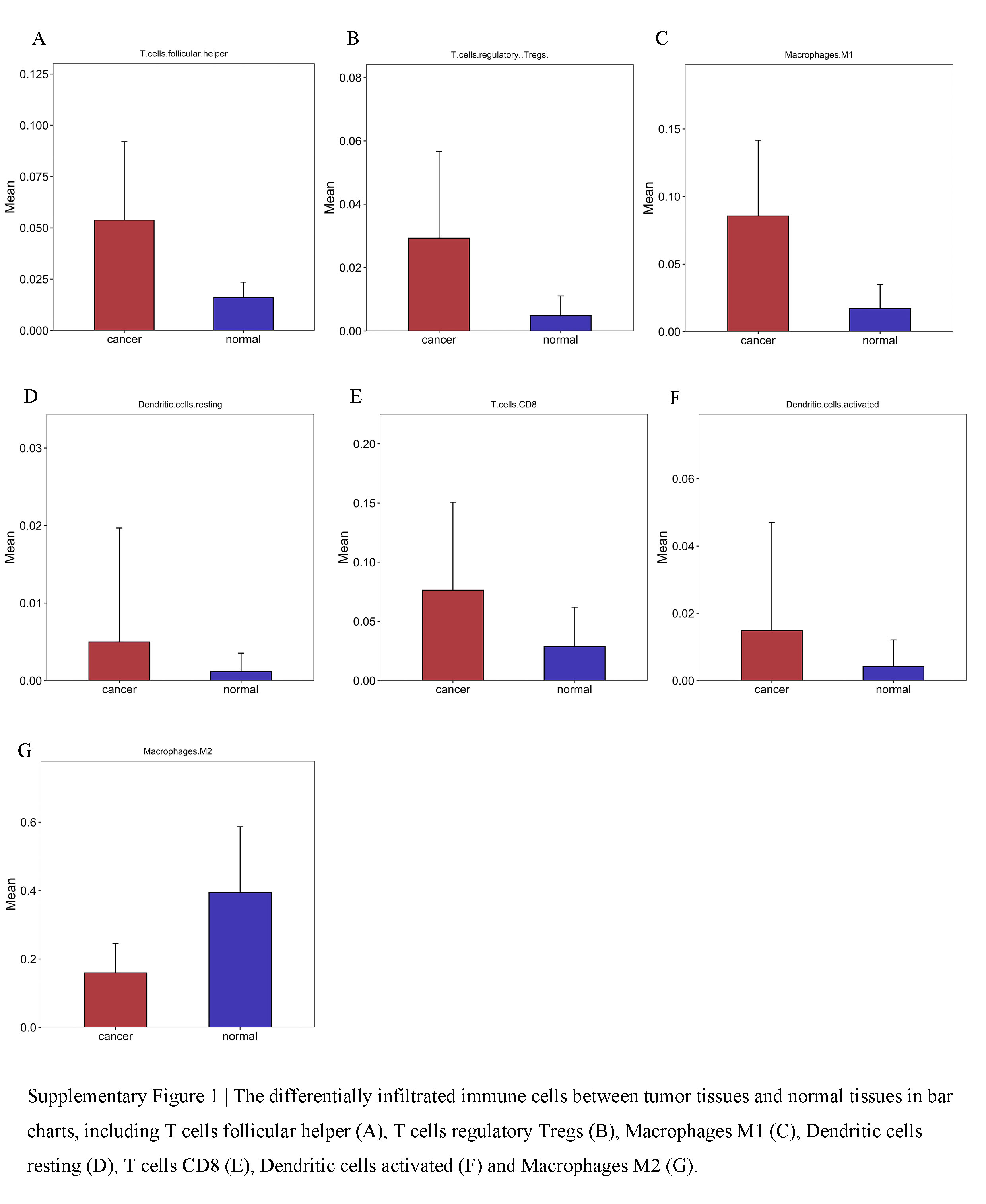

Supplement: Supplementary file 6 [file Image_1.JPEG]

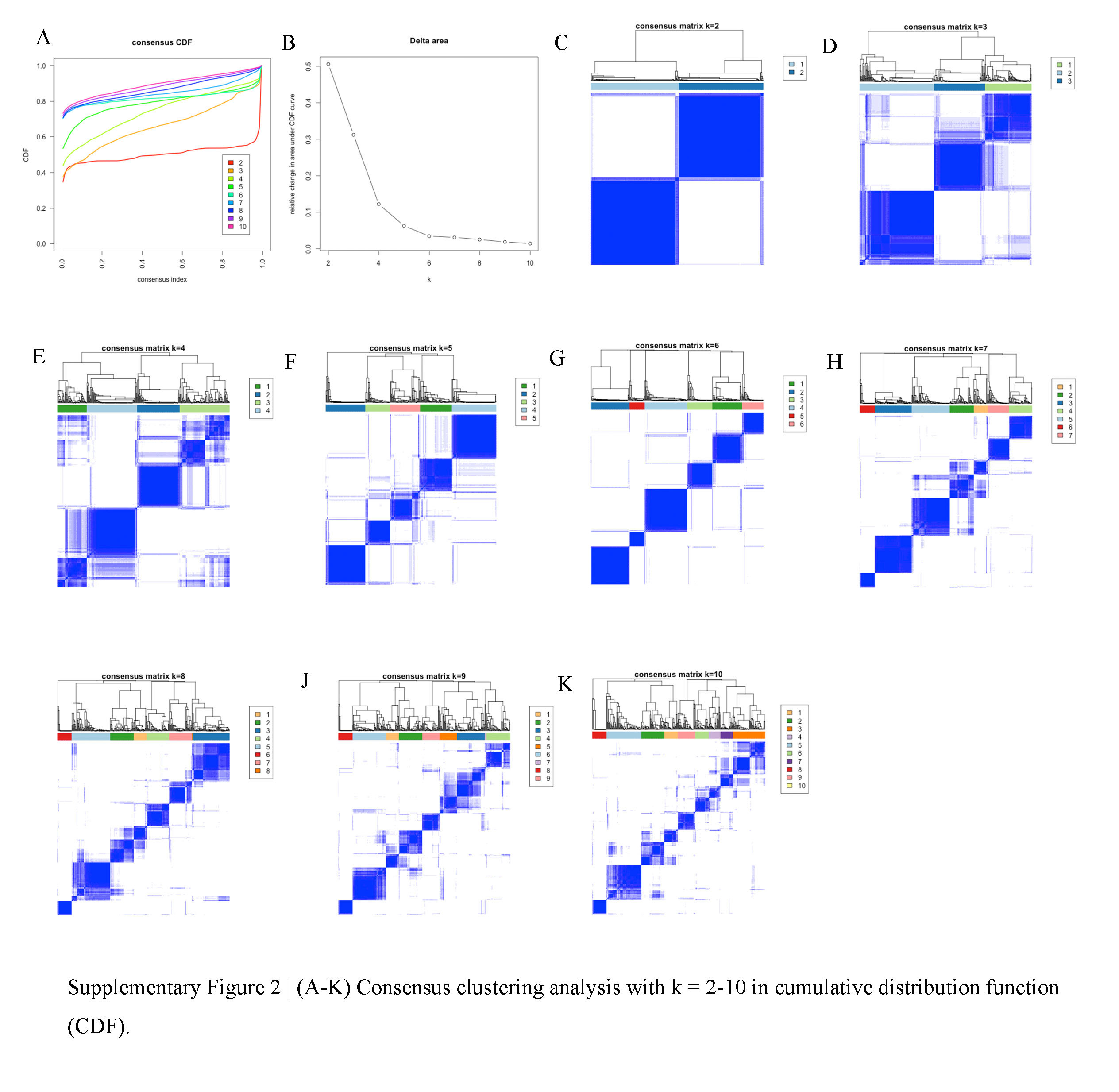

Supplement: Supplementary file 7 [file Image_2.JPEG]
